# Channel Estimation for Intelligent Reflecting Surface Empowered Coal Mine Wireless Communication Systems

**DOI:** 10.3390/e27090932

**Published:** 2025-09-04

**Authors:** Yang Liu, Kaikai Guo, Xiaoyue Li, Bin Wang, Yanhong Xu

**Affiliations:** School of Communication and Information Engineering, Xi’an University of Science and Technology, Xi’an 710054, China; 23207223044@stu.xust.edu.cn (K.G.); 23207040025@stu.xust.edu.cn (X.L.); wangbin@mail.xidian.edu.cn (B.W.); yhxu@xust.edu.cn (Y.X.)

**Keywords:** coal mine wireless communication system, channel model, channel estimation, intelligent reflecting surface

## Abstract

The confined space of coal mines characterized by curved tunnels with rough surfaces and a variety of deployed production equipment induces severe signal attenuation and interruption, which significantly degrades the accuracy of conventional channel estimation algorithms applied in coal mine wireless communication systems. To address these challenges, we propose a modified Bilinear Generalized Approximate Message Passing (mBiGAMP) algorithm enhanced by intelligent reflecting surface (IRS) technology to improve channel estimation accuracy in coal mine scenarios. Due to the presence of abundant coal-carrying belt conveyors, we establish a hybrid channel model integrating both fast-varying and quasi-static components to accurately model the unique propagation environment in coal mines. Specifically, the fast-varying channel captures the varying signal paths affected by moving conveyors, while the quasi-static channel represents stable direct links. Since this hybrid structure necessitates an augmented factor graph, we introduce two additional factor nodes and variable nodes to characterize the distinct message-passing behaviors and then rigorously derive the mBiGAMP algorithm. Simulation results demonstrate that the proposed mBiGAMP algorithm achieves superior channel estimation accuracy in dynamic conveyor-affected coal mine scenarios compared with other state-of-the-art methods, showing significant improvements in both separated and cascaded channel estimation. Specifically, when the NMSE is $10^{-3}$, the SNR of mBiGAMP is improved by approximately 5 dB, 6 dB, and 14 dB compared with the Dual-Structure Orthogonal Matching Pursuit (DS-OMP), Parallel Factor (PARAFAC), and Least Squares (LS) algorithms, respectively. We also verify the convergence behavior of the proposed mBiGAMP algorithm across the operational signal-to-noise ratios range. Furthermore, we investigate the impact of the number of pilots on the channel estimation performance, which reveals that the proposed mBiGAMP algorithm consumes fewer number of pilots to accurately recover channel state information than other methods while preserving estimation fidelity.

## 1. Introduction

Coal mines, as a vertical industrial application for B5G/6G wireless communication networks, face significant challenges due to the intricate layout of tunnels, the rough surfaces of tunnel walls, and the various devices deployed for production demands [1]. These unfavorable factors result in signal attenuation, obstruction, distortion, and multipath interference, which further severely affects the stability and reliability for the coal mine wireless communication system and causes potential safety risks [2,3]. Channel estimation is a critical component to ensure the overall quality of the wireless communication system. Currently, channel estimation for coal mine wireless communication systems typically relies on classical algorithms such as least squares (LS) and minimum mean square error (MMSE). Specially, an improved MMSE algorithm was proposed in [4], which achieves comparable estimation accuracy with reduced computational complexity. However, it requires a substantial amount of pilot overhead, which is impractical in harsh environmental conditions. To further enhance channel estimation performance in coal mines, an improved super resolution convolutional network (SRCNN)-based algorithm was introduced in [5]. This approach improves estimation accuracy while lowering pilot overhead. Nevertheless, the channel model used in [5] is based on a simplified Rayleigh fading model, which does not adequately reflect the complex propagation conditions in coal mines characterized by numerous tunnel bends and extensive transportation equipment. Therefore, there is an urgent need for new wireless communication technologies that are low power, cost-effective, and highly reliable to improve the accuracy of channel estimation and achieve the reliable signal transmission in coal mines.

Intelligent reflecting surface (IRS) technology is considered as one of the potential key technologies for 6G due to its advantages of low cost, low power consumption, ease of deployment, and scalability. IRS technology establishes a new paradigm for intelligent programmable wireless environments, enabling the manipulation of signal propagation directions, signal enhancement, or interference suppression to achieve exceptional communication performance in three-dimensional space [6,7]. Due to the low cost and ease of deployment of IRS, it is expected to be applied in coal mines to address issues of signal blockage and interference caused by obstacles, such as large production equipment and bends in tunnels, and thereby enhance the performance [8,9]. However, direct CSI acquisition in IRS-aided wireless systems is hindered by the IRS’s passive elements, which lack the processing functionality needed to sense and estimate the CSI.

Currently, in IRS-assisted wireless communication systems, various strategies have been proposed to effectively address the channel estimation problem. However, most existing research on channel estimation focused on the ground environments. Specifically, a binary reflection method for a single-user IRS-assisted communication system was proposed in [10], but its training overhead is proportional to the size of IRS, which is often impractical. The work in [11] extended this to multi-user systems by leveraging the sparsity of the cascaded channel and formulating the estimation as a compressed sensing (CS) problem, which can achieve accurate channel estimation with lower training overhead. Since the channel estimation problem can be approximated as a nonlinear mapping from the received signal to the channel information, deep learning offers strong potential for reducing training overhead and time. In [12,13], deep learning-based channel estimation schemes using convolutional neural networks (CNNs) and skip connection attention (SC-attention) networks were proposed to achieve improved channel estimation performance, but a large amount of training data are required in deep learning based algorithms. Unlike the above cascaded-channel estimation approaches, a parallel factor (PARAFAC)-based method was proposed in [14] to separately estimate the user-to-IRS and IRS-to-BS channels using a small number of pilot signals, but the computational complexity is relatively high. To further improve the performance of channel estimation, ref. [15] introduced the approximate message passing (AMP) algorithm to achieve both improved channel estimation accuracy and reduced computational complexity. Building on this, ref. [16] divided the AMP process into three distinct stages and utilized the widely used expected propagation approximation (EPA) methodology and a novel reassembling technology to further improve the performance. However, all these AMP-based studies assume simplified channel models that fail to capture dynamic interference caused by mining equipment (e.g., coal conveyors), which significantly alter signal propagation behavior in coal mines. Therefore, existing AMP algorithm research cannot be directly applied to channel estimation in coal mine environments.

To improve the channel estimation accuracy in coal mine wireless communication systems, we propose a computationally efficient modified bilinear generalized AMP (mBiGAMP) algorithm combined with IRS technology. The main contributions of this paper are summarized as follows.

- Due to the presence of abundant coal-carrying belt conveyors, we establish a hybrid channel model integrating both fast-varying and quasi-static components to accurately model the unique propagation environment in coal mines. Specifically, the fast-varying channel captures the varying signal paths affected by moving conveyors, while the quasi-static channel represents remains stationary links for a certain period of time.
- We derive the mBiGAMP algorithm based on the proposed channel model for the IRS-assisted coal mine wireless communication system. Two additional factor nodes and variable nodes are introduced to characterize the distinct message-passing behaviors and the posterior mean estimation on the factor graph is computed using the sum-product algorithm.
- We analyze the computational complexity of the proposed mBiGAMP algorithm and compare it with existing state-of-the-art methods to demonstrate that it has lower computational complexity. Simulation results first examine the convergence behavior of mBiGAMP. The mBiGAMP algorithm is then compared against several benchmarks—including the PARAFAC [17], dual-structure orthogonal matching pursuit (DS-OMP) [18], and LS algorithms—under various system configurations and in the presence of phase noise. The results confirm that the proposed scheme achieves favorable performance across all considered scenarios.

The rest of the paper is structured as follows. Section 2 describes the system model. Section 3 presents the factor graph formulation and the proposed message passing algorithm, together with additional approximations to reduce computational complexity. Section 4 reports numerical results for the proposed scheme. Finally, Section 5 concludes our work.

## 2. System Model

In this paper, we consider that BS and IRS are fixed post-deployment and the miners exhibit low mobility, which means that the links between them evolve very slowly over a coherence interval and they can be referred to as quasi-static channels. In addition, a minority of propagation paths experience rapid fluctuations induced by interactions with moving conveyor belts, which can be referred to as fast-varying channels. On this basis, we establish a hybrid channel model integrating with quasi-static and fast-varying channels.

**Figure 1:**
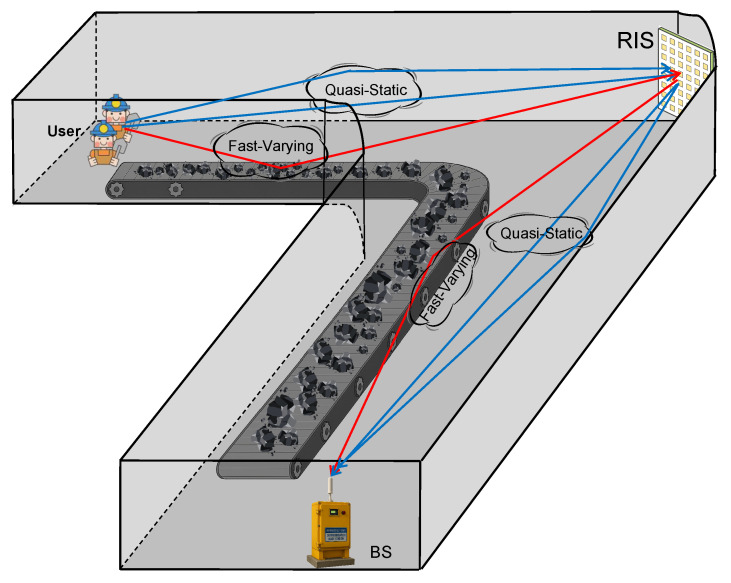
IRS-assisted coal mine wireless communication system model.

The specific IRS-assisted wireless communication system in coal mines is illustrated in Figure 1, where an L-shaped model is employed. We consider an uplink communication scenario in which *K* single-antenna users simultaneously communicate with the BS equipped with *M* antennas. To mitigate line-of-sight (LoS) blockage induced by tunnel bends in the coal mine, an IRS with *N* phase-shift elements is placed at turning points to facilitate communication between the users and the BS. The BS employs a uniform linear array (ULA), and the passive reflecting elements in the IRS are configured as an $N_{1}\times N_{2}$ uniform rectangular array (URA) with $N_{1}N_{2}=N$.

The coefficient channel vectors/matrix of the *k*th user to IRS and IRS to BS are denoted by $h_{\mathrm{UR},k}\in C^{N\times 1}$ and $H_{\mathrm{RB}}\in C^{M\times N}$, respectively. Further, we define the reflection coefficient channel matrix as $\Phi =\mathrm{diag}\left(\phi \right)\in C^{N\times N}$, where $\phi ={\left[\phi_{1},\phi_{2},\ldots ,\phi_{N}\right]}^{T}\in C^{N\times 1},$ and $\phi_{n}=\beta_{n}e^{j\theta_{n}}$ is the reconfigurable reflection coefficient on the *n*th reflective element. Here, $\beta_{n}\in \left(0,1\right]$ denotes the amplitude coefficient; $\theta_{n}\in \left[0,2\pi \right)$ denotes the phase shift of the *n*th IRS element. To facilitate IRS channel estimation, each user simultaneously transmits the training sequence with length *L* to the BS assisted by IRS. We denote the training sequence of the *k*th user as $x_{k}={\left[\begin{array}{c} x_{k1},\cdots ,x_{kl},\cdots ,x_{kL} \end{array}\right]}^{T}$, where $x_{kl}$ is the training symbol of the *k*th user in time slot *l*.

The received signal at the BS at time slot *l* can be written as

(1) $$y_{0}\left(l\right)=\sum_{k=1}^{K}\left(H_{\mathrm{RB}}\Phi h_{\mathrm{UR},k}\right)x_{kl}+n\left(l\right),\;1\leq l\leq L$$

where $n\left(l\right)\in C^{M\times 1}$ is an AWGN vector following the distribution $\mathrm{CN}\;\left(n\left(l\right);0,\;\tau_{N}I_{M}\right)$.

### 2.1. Channel Model

To characterize the quasi-static and fast-varying components of the channel, we model the channel vectors scaled by the Rician factor from the *k*th user to IRS as

(2) $$h_{\mathrm{UR},k}=\sqrt{\frac{\kappa }{\kappa +1}}{\bar{h}}_{\mathrm{UR},k}+\sqrt{\frac{1}{\kappa +1}}{\tilde{h}}_{\mathrm{UR},k},$$

where ${\bar{h}}_{\mathrm{UR},k}$ and ${\tilde{h}}_{\mathrm{UR},k}$ represent the quasi-static and fast-varying channel components from the *k*th user to IRS, respectively. The parameter κ denotes the Rician factor, which determines the proportion of these two components.

Similarly, the channel matrix from IRS to BS is given by

(3) $$H_{\mathrm{RB}}=\sqrt{\frac{\kappa }{\kappa +1}}{\bar{H}}_{\mathrm{RB}}+\sqrt{\frac{1}{\kappa +1}}{\tilde{H}}_{\mathrm{RB}}$$

where ${\bar{H}}_{\mathrm{RB}}$ and ${\tilde{H}}_{\mathrm{RB}}$ represent the quasi-static and fast-varying channel components from IRS to BS, respectively.

For the quasi-static channel component, to better match the actual signal transmission characteristics in the coal mine tunnel, we consider the multipath effects caused by the rough surface and abundant scatterers in the coal mine tunnel and incorporate them as multipath components into the quasi-static channel component. Specifically, as shown in [19], the channel vector from the *k*th user to the IRS in the quasi-static component can be expressed as the weighted sum of the line-of-sight (LoS) path and multiple non-line-of-sight (NLoS) paths. Therefore,

(4) $${\bar{h}}_{\mathrm{UR},k}\left(t,\tau \right)=\sqrt{\frac{\kappa_{1}}{\kappa_{1}+1}}{\bar{h}}_{\mathrm{UR},k}^{L}\left(t,\tau \right)+\sqrt{\frac{1}{\kappa_{1}+1}}{\bar{h}}_{\mathrm{UR},k}^{NL}\left(t,\tau \right)$$

where $\kappa_{1}$ is the Rice factor used to control the size of the LoS path component and the NLoS paths.

The LoS component ${\bar{h}}_{\mathrm{UR},k}^{L}\left(t,\tau \right)$ is given by

(5) $${\bar{h}}_{\mathrm{UR},k}^{L}\left(t,\tau \right)=e^{j2\pi \tau_{\mathrm{UR},k}^{L}f_{c}}\;\delta \left(\tau -\tau_{\mathrm{UR},k}^{L}\right)$$

where $\tau_{\mathrm{UR},k}^{L}$ is the propagation delay of LoS component and is computed by $\tau_{\mathrm{UR},k}^{L}=D_{\mathrm{UR},k}/c$, where $D_{\mathrm{UR},k}$ is the distance between the *k*th user and IRS, and *c* represents the speed of light.

The NLoS component ${\bar{h}}_{\mathrm{UR},k}^{NL}\left(t,\tau \right)$ is given by

(6) $${\bar{h}}_{\mathrm{UR},k}^{NL}\left(t,\tau \right)=\sum_{n=1}^{N_{\mathrm{UR},k}}\sum_{m=1}^{M_{\mathrm{UR},k}}\sqrt{P_{m}}\;e^{j2\pi f_{c}\tau_{m}}\;\delta \left(\tau -\tau_{m}\right)$$

where $N_{\mathrm{UR},k}$ is the number of clusters and $M_{\mathrm{UR},k}$ is the number of rays in the *n*th cluster; $f_{c}$ is the operating frequency; $P_{m}$ and $\tau_{m}$ denote the normalized power and delay of the *m*th ray in the *n*th cluster between the *k*th user and the IRS, respectively. The propagation delay $\tau_{m}$ is calculated as $\tau_{m}=\frac{D_{m}}{c}+{\tilde{\tau }}_{m}$, where $D_{m}$ represents the travel distance of NLoS component and ${\tilde{\tau }}_{m}$ means the propagation delay of virtual link.

Similarly, the quasi-static component from the IRS to the BS can be expressed as

(7) $${\bar{H}}_{\mathrm{RB}}\left(t,\tau \right)=\sqrt{\frac{\kappa_{1}}{\kappa_{1}+1}}\;{\bar{H}}_{\mathrm{RB}}^{L}\left(t,\tau \right)+\sqrt{\frac{1}{\kappa_{1}+1}}\;{\bar{H}}_{\mathrm{RB}}^{NL}\left(t,\tau \right)$$

where

(8) $${\bar{H}}_{\mathrm{RB}}^{L}\left(t,\tau \right)=e^{j2\pi \tau_{\mathrm{RB}}^{L}f_{c}}\;\delta \left(\tau -\tau_{\mathrm{RB}}^{L}\right),$$

(9) $${\bar{H}}_{\mathrm{RB}}^{NL}\left(t,\tau \right)=\sum_{n=1}^{N_{\mathrm{RB}}}\sum_{m=1}^{M_{\mathrm{RB}}}\sqrt{P_{m}^{\prime }}\;e^{j2\pi f_{c}\tau_{m}^{\prime }}\;\delta \left(\tau -\tau_{m}^{\prime }\right)$$

where $N_{\mathrm{RB}}$ is the number of clusters and $M_{\mathrm{RB}}$ is the number of rays in the *n*th cluster; $P_{m}^{\prime }$ and $\tau_{m}^{\prime }$ denote the normalized power and delay of the *m*th ray in the *n*th cluster between the IRS and the BS, respectively.

For the fast-varying channel component, we consider the geometric channel model proposed in [20], which can accurately describe the propagation process of the signal in space including reflection, diffraction, and refraction. Therefore, the channel vectors from the *k*th user to IRS can be expressed as

(10) $${\tilde{h}}_{\mathrm{UR},k}=\sqrt{\frac{N}{{\tilde{P}}_{k}}}\sum_{p=1}^{{\tilde{P}}_{k}}\alpha_{\tilde{p}}a_{R}\left(\psi_{p},\sigma_{p}\right),$$

where ${\tilde{P}}_{k}$ represent the numbers of fast-varying paths from the *k*th user to IRS; $\alpha_{\tilde{p}}$ is the corresponding complex-valued channel coefficient, following the Nakagami-*m* distribution; $\psi_{p}$ and $\sigma_{p}$ are the azimuth and elevation angles of arrival (AoA) corresponding to the *p*th path with respect to IRS; $a_{R}$ is the steering vector associated with IRS antenna geometry given by

(11) $$a_{R}\left(\psi ,\sigma \right)=f_{N_{2}}\left(-\cos\left(\sigma \right)\cos\left(\psi \right)\right)⊗f_{N_{1}}\left(\cos\left(\sigma \right)\sin\left(\psi \right)\right),$$

where ⊗ represents the Kronecker product, and

(12) $$f_{L}\left(x\right)=\frac{1}{\sqrt{L}}{\left[\begin{array}{c} 1,e^{-j\frac{2\pi }{\lambda }dx},\ldots ,e^{-j\frac{2\pi }{\lambda }d\left(L-1\right)x} \end{array}\right]}^{T}.$$

In (12), λ denotes the carrier wavelength and *d* denotes the distance between any two adjacent antennas. Here, we set d/λ=1/2 for simplicity.

Similarly, the channel matrix from IRS to BS can be expressed as

(13) $${\tilde{H}}_{\mathrm{RB}}=\sqrt{\frac{MN}{{\tilde{P}}_{\mathrm{RB}}}}\sum_{p=1}^{{\tilde{P}}_{\mathrm{RB}}}{\hat{\alpha }}_{\tilde{P}}a_{B}\left(\phi_{p}\right)a_{R}^{H}\left({\hat{\psi }}_{p},{\hat{\sigma }}_{p}\right),$$

where ${\tilde{P}}_{RB}$ represent the numbers of quasi-static and fast-varying paths from IRS to BS; ${\hat{\alpha }}_{\tilde{p}}$ is the corresponding complex-valued channel coefficients, following the Nakagami-*m* distribution; $\phi_{p}$ is the AoA corresponding to the *p*th path with respect to BS; ${\hat{\psi }}_{p}$ and ${\hat{\sigma }}_{p}$ are the azimuth and elevation angle-of-departure (AoD) corresponding to the *p*th path with respect to IRS; $a_{B}$ is the steering vector associated with BS antenna geometry and can be expressed as

(14) $$a_{B}\left(\phi \right)=f_{M}\left(\sin\left(\phi \right)\right).$$

### 2.2. Angular Domain Channel Representation

We assume that the quasi-static channel remains stationary for a certain period of time. Therefore, before channel estimation, we assume that the quasi-static channel is known. Even if some errors occur in the known quasi-static component, they will be absorbed by the fast-varying component and will not affect the final estimation accuracy. Hence, treating the quasi-static channel as known here is without loss of generality.

As shown above, the fast-varying component matrix contains only a limited number of paths, exhibiting sparsity in the angle-domain channel representation. By exploiting this angular-domain sparsity, the performance of channel estimation can be significantly improved. Following [21], we employ two sampling grids ξ with length $N_{1}^{\prime }$ ($N_{1}^{\prime }\geq N_{1}$) and Ψ with length $N_{2}^{\prime }$ ($N_{2}^{\prime }\geq N_{2}$) to respectively discretize the sets $\left\{\cos\left(\sigma_{p}\right)\sin\left(\psi_{p}\right)\right\}$ and $\left\{-\cos\left(\sigma_{p}\right)\cos\left(\psi_{p}\right)\right\}$ in (11). Then, we express the fast-varying component vectors ${\tilde{h}}_{\mathrm{UR},k}$ in the angular domain as

(15) $$\sqrt{\frac{1}{\kappa +1}}{\tilde{h}}_{\mathrm{UR},k}=\left(A_{R,v}⊗A_{R,h}\right)g_{k}$$

where $A_{R,h}≜\left[\begin{array}{c} f_{N_{1}}\left(\xi_{1}\right),\cdots ,f_{N_{1}}\left(\xi_{N_{1}^{\prime }}\right) \end{array}\right]\in C^{N_{1}\times N_{1}^{\prime }}$ and $A_{R,v}≜\left[\begin{array}{c} f_{N_{2}}\left(\Psi_{1}\right),\cdots ,f_{N_{2}}\left(\Psi_{N_{2}^{\prime }}\right) \end{array}\right]\in C^{N_{2}\times N_{2}^{\prime }}$ are the over-complete horizontal and vertical array responses, respectively; $g_{k}\in C^{N^{\prime }\times 1}$ denote the angular-domain channel coefficient vector of ${\tilde{h}}_{\mathrm{UR},k}$, with $N^{\prime }=N_{1}^{\prime }N_{2}^{\prime }$. Because the number of propagation paths ${\tilde{P}}_{k}$ is small, only a limited subset of entries in $g_{k}$ are nonzero, each corresponding to one path. Hence, $g_{k}$ is a sparse vector.

Similarly, we adopt an oversampled angular grid ϑ of size $M^{\prime }$ ($M^{\prime }\geq M$) to discretize $\left\{\sin\left(\phi_{p}\right)\right\}$ in (14). Then, we express the fast-varying component matrix ${\tilde{H}}_{\mathrm{RB}}$ in the angular domain as    

(16) $$\sqrt{\frac{1}{\kappa +1}}{\tilde{H}}_{\mathrm{RB}}=A_{B}SA_{R}^{H}$$

where $A_{B}≜\left[\begin{array}{c} f_{M}\left(\vartheta_{1}\right),\cdots ,f_{M}\left(\vartheta_{M^{\prime }}\right) \end{array}\right]\in C^{M\times M^{\prime }}$ is an over-complete array response; $S\in C^{M^{\prime }\times N^{\prime }}$ denote the angular domain channel coefficient matrix of ${\tilde{H}}_{\mathrm{RB}}$; $A_{R}=\left(A_{R,v}⊗A_{R,h}\right)\in C^{N\times N^{\prime }}$. Because the number of propagation paths ${\tilde{P}}_{\mathrm{RB}}$ is small, only a limited subset of entries in S are nonzero, each corresponding to a distinct path. Hence, S is a sparse matrix.

Based on the representations of (2), (3), (15), and (16) above, Equation (1) can be recast as

(17) $$\begin{array}{cc} Y & =\left(\sqrt{\frac{\kappa }{\kappa +1}}{\bar{H}}_{\mathrm{RB}}+A_{B}SA_{R}^{H}\right)\Phi \left(\sqrt{\frac{\kappa }{\kappa +1}}{\bar{H}}_{\mathrm{UR}}+A_{R}G\right)X+N\\ & =\left(H_{0}+A_{B}SA_{R}^{H}\right)\Phi \left(H_{1}+A_{R}G\right)X+N \end{array}$$

where $X={\left[\begin{array}{c} x_{1},\cdots ,x_{K} \end{array}\right]}^{T}\in C^{K\times L}$; $N=\left[\begin{array}{c} n(1),\cdots ,n(L) \end{array}\right]\in C^{M\times L}$; $Y=\left[\begin{array}{c} y_{0}\left(0\right),\cdots ,y_{0}\left(L\right) \end{array}\right]\in C^{M\times L}$; $G=\left[\begin{array}{c} g_{1},\cdots ,g_{K} \end{array}\right]\in C^{N^{\prime }\times K}$; $H_{0}=\sqrt{\;\kappa /(\kappa +1)}\cdot {\bar{H}}_{\mathrm{RB}}$; $H_{1}=\sqrt{\;\kappa /(\kappa +1)}\cdot {\bar{H}}_{\mathrm{UR}}$. 

Based on Y in (17), the BS seeks to estimate the channel matrices S and G using the known training matrix X. This is discussed in the following sections.

## 3. Problem Formulation and Message Passing Algorithm

### 3.1. Problem Formulation

In this section, we derive, within a Bayesian framework, the posterior mean estimates of S and G conditioned on Y. Subsequently, the sum–product algorithm (SPA) [22] is applied to obtain the message-passing updates.

Define $W=H_{0}+A_{B}SA_{R}^{H}\in C^{M\times N}$, $F=\Phi \left(H_{1}+A_{R}G\right)\in C^{N\times K}$ and $Z=\mathrm{WF}\in C^{M\times K}$. The auxiliary variable $Q=\mathrm{ZX}\in C^{M\times L}$ is introduced. Under the AWGN assumption, we have

(18) $$p\left(Y|Q\right)=\prod_{m=1}^{M}\prod_{l=1}^{L}\mathrm{CN}\left(y_{ml};q_{ml},\tau_{N}\right).$$

According to Bayes’ theorem, the posterior can be expressed as

(19) $$p\left(S,G|Y\right)=\frac{1}{p(Y)}p\left(Y|S,G\right)p\left(S\right)p\left(G\right),$$

where p(Y)=∫p(Y∣S,G)p(S)p(G)dSdG. The prior distributions p(S) and p(G) follow the Nakagami-*m* distribution as detailed in [23], with sparsity parameters $\lambda_{S}$ and $\lambda_{G}$, respectively. Using the minimum mean square error (MMSE) criterion, the MMSE estimators $\hat{S}$ and $\hat{G}$ of S and G solve for

(20) $$\min_{\hat{S}}E\left\{∥S-\hat{S}{∥}_{F}^{2}\right\},\;\min_{\hat{G}}E\left\{∥G-\hat{G}{∥}_{F}^{2}\right\}.$$

According to Bayes’ rule and the first order optimal condition, the channel estimates $\hat{S}=\left[{\hat{s}}_{m^{\prime }n^{\prime }}\right]$ and $\hat{G}=\left[{\hat{g}}_{n^{\prime }k}\right]$ are given by the following posterior mean estimators:

(21) $${\hat{s}}_{m^{\prime }n^{\prime }}=E\left\{s_{m^{\prime }n^{\prime }}|Y\right\},\;{\hat{g}}_{n^{\prime }k}=E\left\{g_{n^{\prime }k}|Y\right\}$$

where the expectations are taken with respect to the marginal posterior distributions $p(s_{m^{\prime }n^{\prime }}|Y)$ and $p(g_{n^{\prime }k}|Y)$, respectively.

### 3.2. Factor Graph Representation and Message Passing Algorithm

The exact computation of $\hat{S}$ and $\hat{G}$ is generally intractable because the marginalization entails high-dimensional integrals over S and G. In the following, we derive an approximate solution within a message-passing framework.

Using the relations among Y,Z,W,F,G, and S, the posterior p(G,S∣Y) can be expressed as

(22) $$\begin{array}{cc} p(S,G|Y) & =\frac{1}{p(Y)}\left(\prod_{m=1}^{M}\prod_{l=1}^{L}p\left(y_{ml}|z_{mk},\forall k\right)\right)\left(\prod_{m=1}^{M}\prod_{k=1}^{K}p\left(z_{mk}|w_{mn},f_{nk},\forall n\right)\right)\\ & \times \left(\prod_{m=1}^{M}\prod_{n=1}^{N}p\left(w_{mn}|s_{m^{\prime }n^{\prime }},\forall m^{\prime },n^{\prime }\right)\right)\left(\prod_{m^{\prime }=1}^{M^{\prime }}\prod_{n^{\prime }=1}^{N^{\prime }}p\left(s_{m^{\prime }n^{\prime }}\right)\right)\\ & \times \left(\prod_{n=1}^{N}\prod_{k=1}^{K}p\left(f_{nk}|g_{nk}\right)\right)\left(\prod_{n=1}^{N^{\prime }}\prod_{k=1}^{K}p\left(g_{nk}\right)\right). \end{array}$$

**Figure 2:**
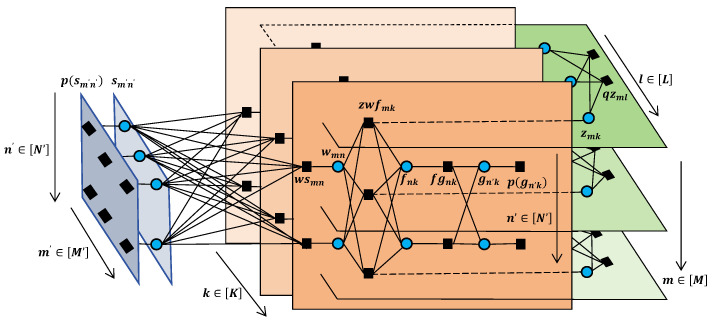
Factor graph for message passing.

We build a factor graph corresponding to (22) and employ the canonical message-passing algorithm to obtain approximate solutions for the estimators in (21). The factor graph is shown in Figure 2.

Since this hybrid structure necessitates an augmented factor graph, we introduce two additional factor nodes and variable nodes to characterize the distinct message-passing behaviors. In the factor graph, $M=M^{\prime }=K=3$ and $N=N^{\prime }=L=2$. The blue circles represent variable nodes, and the black squares represent factor nodes. To apply the SPA, we define the following message notation: $\Delta_{a\to b}^{i}\left(\cdot \right)$ represents the message passed from node *a* to *b* during the *i*th iteration, and $\Delta_{v}^{i}\left(\cdot \right)$ represents the edge message calculated at variable node *v* during the *i*th iteration.

By the SPA rule, the messages are as follows:

(1) The message passing expressions between variable nodes $\left\{z_{mk}\right\}$ and factor nodes $\left\{qz_{ml}\right\}$ are as follows:
  (23) $$\Delta_{qz_{ml}\to z_{mk}}^{i}\left(z_{mk}\right)\propto \int \mathrm{CN}\left(y_{ml};\sum_{k=1}^{K}z_{mk}x_{kl},\tau_{N}\right)\prod_{j\neq k}\Delta_{z_{mj}\to qz_{ml}}^{i}\left(z_{mj}\right)\;dz_{mj},$$
  (24) $$\Delta_{z_{mk}\to qz_{ml}}^{i+1}\left(z_{mk}\right)\propto P_{z_{mk}}^{i}\left(z_{mk}\right)\prod_{j\neq l}\Delta_{qz_{mj}\to z_{mk}}^{i}\left(z_{mk}\right),$$
  where the auxiliary distribution $P_{z_{mk}}^{i}\left(z_{mk}\right)$ is defined as
  (25) $$P_{z_{mk}}^{i}\left(z_{mk}\right)\propto \int p\left(z_{mk}|w_{mn},f_{nk},\forall n\right)\prod_{n=1}^{N^{\prime }}\left(\Delta_{w_{mn}\to zwf_{mk}}^{i}\left(w_{mn}\right)\Delta_{f_{nk}\to zwf_{mk}}^{i}\left(f_{nk}\right)dw_{mn}df_{nk}\right),$$
  the marginal distribution of the variable node $z_{mk}$ is given by
  (26) $$\Delta_{z_{mk}}^{i+1}\left(z_{mk}\right)\propto P_{z_{mk}}^{i}\left(z_{mk}\right)\prod_{l=1}^{L}\Delta_{qz_{ml}\to z_{mk}}^{i}\left(z_{mk}\right).$$
(2) The message passing expressions between variable nodes $\left\{f_{nk}\right\}$ and factor nodes $\left\{zwf_{mk}\right\}$ are as follows:
  (27) $$\begin{array}{cc} \Delta_{zwf_{mk}\to f_{nk}}^{i}\left(f_{nk}\right) & \propto \int dz_{mk}\;p\left(z_{mk}|w_{mn},f_{nk}\right)\prod_{l=1}^{L}\Delta_{qz_{mt}\to z_{mk}}^{i}\left(z_{mk}\right)\\ & \times \prod_{j\neq n}\left(\Delta_{f_{jk}\to zwf_{mk}}^{i}\left(f_{jk}\right)\;df_{jk}\right)\prod_{n=1}^{N}\left(\Delta_{w_{mn}\to zwf_{mk}}^{i}\left(w_{mn}\right)\;dw_{mn}\right), \end{array}$$
  (28) $$\Delta_{f_{nk}\to zwf_{mk}}^{i+1}\left(f_{nk}\right)\propto P_{f_{nk}}^{i}\left(f_{nk}\right)\prod_{j\neq m}\Delta_{zwf_{mk}\to f_{nk}}^{i}\left(f_{nk}\right),$$
  where the auxiliary distribution $P_{f_{nk}}^{i}\left(f_{nk}\right)$ is defined as
  (29) $$P_{f_{nk}}^{i}\left(f_{nk}\right)\propto \int \prod_{n^{\prime }=1}^{N^{\prime }}\prod_{k=1}^{K}\left(\Delta_{g_{n^{\prime }k}\to fg_{nk}}^{i}\left(g_{n^{\prime }k}\right)\;dg_{n^{\prime }k}\right)\;p\left(f_{nk}|g_{n^{\prime }k}\right),$$
  the marginal distribution of the variable node $f_{nk}$ is given by
  (30) $$\Delta_{f_{nk}}^{i+1}\left(f_{nk}\right)\propto P_{f_{nk}}^{i}\left(f_{nk}\right)\prod_{m=1}^{M}\Delta_{zwf_{mk}\to f_{nk}}^{i}\left(f_{nk}\right).$$
(3) The message passing expressions between variable nodes $\left\{w_{mn}\right\}$ and factor nodes $\left\{zwf_{mk}\right\}$ are as follows:
  (31) $$\begin{array}{cc} \Delta_{zwf_{mk}\to w_{mn}}^{i}\left(w_{mn}\right) & \propto \int dz_{mk}\;p\left(z_{mk}|w_{mn},f_{nk}\right)\prod_{l=1}^{L}\Delta_{qz_{ml}\to z_{mk}}^{i}\left(z_{mk}\right)\\ & \times \prod_{j\neq n}\left(\Delta_{w_{mj}\to zwf_{mk}}^{i}\left(w_{mj}\right)\;dw_{mj}\right)\prod_{n=1}^{N}\left(\Delta_{f_{nk}\to zwf_{mk}}^{i}\left(f_{nk}\right)\;df_{nk}\right), \end{array}$$
  (32) $$\Delta_{w_{mn}\to zwf_{mk}}^{i+1}\left(w_{mn}\right)\propto P_{w_{mn}}^{i}\left(w_{mn}\right)\prod_{j\neq k}\Delta_{zwf_{mj}\to w_{mn}}^{i}\left(w_{mn}\right),$$
  where the auxiliary distribution $P_{w_{mn}}^{i}\left(w_{mn}\right)$ is defined as
  (33) $$P_{w_{mn}}^{i}\left(w_{mn}\right)\propto \int \prod_{m^{\prime }=1}^{M^{\prime }}\prod_{n^{\prime }=1}^{N^{\prime }}\left(\Delta_{s_{m^{\prime }n^{\prime }}\to ws_{nk}}^{i}\left(s_{m^{\prime }n^{\prime }}\right)\;ds_{m^{\prime }n^{\prime }}\right)\;p\left(w_{mn}|s_{m^{\prime }n^{\prime }}\right),$$
  the marginal distribution of the variable node $w_{mn}$ is given by
  (34) $$\Delta_{w_{mn}}^{i+1}\left(w_{mn}\right)\propto P_{w_{mn}}^{i}\left(w_{mn}\right)\prod_{k=1}^{K}\Delta_{zwf_{mk}\to w_{mn}}^{i}\left(w_{mn}\right).$$
(4) The message passing expressions between variable nodes $\left\{g_{n^{\prime }k}\right\}$ and factor nodes $\left\{fg_{nk}\right\}$ are as follows:
  (35) $$\begin{array}{cc} \Delta_{fg_{nk}\to g_{n^{\prime }k}}^{i}\left(g_{n^{\prime }k}\right) & \propto \int df_{nk}\;p\left(f_{nk}|g_{n^{\prime }k}\right)\prod_{k=1}^{K}\Delta_{zwf_{mk}\to f_{nk}}^{i}\left(f_{nk}\right)\\ \; & \times \prod_{\left(j,l\right)\neq \left(n^{\prime },k\right)}\left(\Delta_{g_{jl}\to fg_{nk}}^{i}\left(g_{jl}\right)\;dg_{jl}\right), \end{array}$$
  (36) $$\Delta_{g_{n^{\prime }k}\to fg_{nk}}^{i+1}\left(g_{n^{\prime }k}\right)\propto p\left(g_{n^{\prime }k}\right)\prod_{\left(j,l\right)\neq \left(n^{\prime },k\right)}\Delta_{fg_{jl}\to g_{n^{\prime }k}}^{i}\left(g_{n^{\prime }k}\right),$$
  the marginal distribution of the variable node $g_{n^{\prime }k}$ is given by
  (37) $$\Delta_{g_{n^{\prime }k}}^{i+1}\left(g_{n^{\prime }k}\right)\propto p\left(g_{n^{\prime }k}\right)\prod_{n=1}^{N}\prod_{k=1}^{K}\Delta_{fg_{nk}\to g_{n^{\prime }k}}^{i}\left(g_{n^{\prime }k}\right).$$
(5) The message passing expressions between variable nodes $\left\{s_{m^{\prime }n^{\prime }}\right\}$ and factor nodes $\left\{ws_{mn}\right\}$ are as follows:
  (38) $$\begin{array}{cc} \Delta_{ws_{mn}\to s_{m^{\prime }n^{\prime }}}^{i}\left(s_{m^{\prime }n^{\prime }}\right) & \propto \int dw_{mn}\;p\left(w_{mn}|s_{m^{\prime }n^{\prime }}\right)\prod_{k=1}^{K}\Delta_{zwf_{mk}\to w_{mn}}^{i}\left(w_{mn}\right)\\ & \times \prod_{\left(j,l\right)\neq \left(m^{\prime }n^{\prime }\right)}\left(\Delta_{s_{jl}\to ws_{mn}}^{i}\left(s_{jl}\right)\;ds_{jl}\right), \end{array}$$
  (39) $$\Delta_{s_{m^{\prime }n^{\prime }}\to ws_{mn}}^{i+1}\left(s_{m^{\prime }n^{\prime }}\right)\propto p\left(s_{m^{\prime }n^{\prime }}\right)\prod_{\left(j,l)\neq (m,n\right)}\Delta_{ws_{jl}\to s_{m^{\prime }n^{\prime }}}^{i}\left(s_{m^{\prime }n^{\prime }}\right),$$
  the marginal distribution of the variable node $s_{m^{\prime }n^{\prime }}$ is given by
  (40) $$\Delta_{s_{m^{\prime }n^{\prime }}}^{i+1}\left(s_{m^{\prime }n^{\prime }}\right)\propto p\left(s_{m^{\prime }n^{\prime }}\right)\prod_{m=1}^{M}\prod_{n=1}^{N}\Delta_{ws_{mn}\to s_{m^{\prime }n^{\prime }}}^{i}\left(s_{m^{\prime }n^{\prime }}\right).$$

### 3.3. Approximation of Message Passing

The messages in (23)–(40) are, in general, intractable to compute because they involve high-dimensional integrals and normalization terms. Accordingly, we approximate the SPA updates in (23)–(40) using the central limit theorem (CLT) and Taylor-series expansions. For notational convenience, the message means and variances are defined in Table 1.

First, we approximate the messages associated with the variable nodes $\left\{z_{mk}\right\}$. We approximate $\prod_{j\neq k}\Delta_{z_{mj}\to qz_{ml}}^{i}\left(z_{mj}\right)$ in (19) by the CLT as a Gaussian random variable with mean ${\hat{q}}_{ml}\left(i\right)-{\hat{z}}_{mk,l}\left(i\right)\;x_{kl}$ and variance $v_{ml}^{q}\left(i\right)-v_{mk,l}^{z}\left(i\right)\;{\left|x_{kl}\right|}^{2}$, where ${\hat{q}}_{ml}\left(i\right)≜\sum_{k=1}^{K}{\hat{z}}_{mk,l}\left(i\right)\;x_{kl}$ and $v_{ml}^{q}\left(i\right)≜\sum_{k=1}^{K}v_{mk,l}^{z}\left(i\right)\;{\left|x_{kl}\right|}^{2}$. Following the steps in [24], we have

(41) $$\prod_{l=1}^{L}\Delta_{qz_{ml}\to z_{mk}}^{i}\left(z_{mk}\right)=\mathrm{CN}(z_{mk};\;{\hat{\varrho }}_{mk}\left(i\right),\;v_{mk}^{\varrho }\left(i\right)),$$

where the mean ${\hat{\varrho }}_{mk}\left(i\right)$ and the variance $v_{mk}^{\varrho }\left(i\right)$ are further specified in (46).

Applying the Fourier inversion theorem in conjunction with a second-order Taylor expansion to (25) yields $P_{z_{mk}}^{i}\left(z_{mk}\right)\approx \mathrm{CN}\left(z_{mk};{\hat{p}}_{mk}^{i}\left(i\right),\nu_{mk}^{p}\left(i\right)\right)$, where

(42) $${\hat{p}}_{mk}\left(i\right)=\sum_{n=1}^{N^{\prime }}{\hat{w}}_{mn,k}\left(i\right){\hat{f}}_{nk,m}\left(i\right),$$

(43) $$v_{mk}^{p}\left(i\right)=\sum_{n=1}^{N^{\prime }}\left({\left|{\hat{w}}_{mn,k}\left(i\right)\right|}^{2}v_{nk,m}^{f}\left(i\right)+v_{mn,k}^{w}\left(i\right){\left|{\hat{f}}_{nk,m}\left(i\right)\right|}^{2}+v_{mn,k}^{w}\left(i\right)v_{nk,m}^{f}\left(i\right)\right).$$

Based on these two approximations, we represent $\Delta_{z_{mk}}^{i+1}$ in (26) as a Gaussian distribution with tractable mean and variance, explicitly given by

(44) $$\Delta_{z_{mk}}^{i+1}\left(z_{mk}\right)=\mathrm{CN}\left(z_{mk};{\hat{z}}_{mk}\left(i+1\right),\nu_{mk}^{z}\left(i+1\right)\right),$$

where

(45) $${\hat{z}}_{mk}\left(i+1\right)=\frac{v_{mk}^{p}\left(i\right)\;{\hat{\varrho }}_{mk}\left(i\right)+{\hat{p}}_{mk}\left(i\right)\;v_{mk}^{\varrho }\left(i\right)}{v_{mk}^{p}\left(i\right)+v_{mk}^{\varrho }\left(i\right)},\;v_{mk}^{z}\left(i+1\right)=\frac{v_{mk}^{p}\left(i\right)\;v_{mk}^{\varrho }\left(i\right)}{v_{mk}^{p}\left(i\right)+v_{mk}^{\varrho }\left(i\right)}.$$

In the above, $v_{mk}^{\varrho }\left(i\right)$ and ${\hat{\varrho }}_{mk}\left(i\right)$ are given in [15] as follows:

(46) $$v_{mk}^{\varrho }\left(i\right)={\left(\sum_{l=1}^{L}v_{ml}^{\gamma }\left(i\right)\;{\left|x_{kl}\right|}^{2}\right)}^{-1},\;{\hat{\varrho }}_{mk}\left(i\right)={\hat{z}}_{mk}\left(i\right)\;+\;v_{mk}^{\varrho }\left(i\right)\;\sum_{t=1}^{T}x_{kl}{\hat{\gamma }}_{ml}\left(i\right),$$

(47) $$v_{ml}^{\gamma }\left(i\right)={\left(v_{ml}^{\varepsilon }\left(i\right)+\tau_{N}\right)}^{-1},\;{\hat{\gamma }}_{ml}\left(i\right)=v_{ml}^{\gamma }\left(i\right)\left(y_{ml}-{\hat{\varepsilon }}_{ml}\left(i\right)\right),$$

where

(48) $$v_{ml}^{\varepsilon }\left(i\right)=\sum_{k=1}^{K}v_{mk}^{z}\left(i\right){\left|x_{kl}\right|}^{2},\;{\hat{\varepsilon }}_{ml}\left(i\right)=\sum_{k=1}^{K}{\hat{z}}_{mk}\left(i\right)x_{kl}-v_{ml}^{\varepsilon }\left(i\right){\hat{\gamma }}_{ml}\left(i-1\right).$$

To reduce complexity further, we note that the only difference between $\Delta_{z_{mk}\to qz_{ml}}^{i+1}$ and $\Delta_{z_{mk}}^{i+1}$ is a single term that vanishes in the large-system limit. Thus, we use the mean and variance of $\Delta_{z_{mk}}^{i+1}$ (i.e., ${\hat{z}}_{mk}\left(i+1\right)$ and $v_{mk}\left(i+1\right)$) to approximate the mean and variance of $\Delta_{z_{mk}\to qz_{ml}}^{i+1}$. Specifically, they are given by

(49) $$\Delta_{z_{mk}\to qz_{ml}}^{i+1}\left(z_{mk}\right)=\mathrm{CN}\left(z_{mk};{\hat{z}}_{mk,l}\left(i+1\right),\nu_{mk,l}^{z}\left(i+1\right)\right),$$

where

(50) $${\hat{z}}_{mk,l}\left(i+1\right)\approx {\hat{z}}_{mk}\left(i+1\right)-v_{mk}^{z}\left(i+1\right)x_{kl}{\hat{\gamma }}_{ml}\left(i\right),$$

(51) $$v_{mk,l}^{z}\left(i+1\right)\approx v_{mk}^{z}\left(i+1\right).$$

Then we approximate the messages associated with the variable nodes $\left\{f_{nk}\right\}$ as follows:

(52) $$\prod_{m=1}^{M}\Delta_{zwf_{mk}\to f_{nk}}^{i}\left(f_{nk}\right)=\mathrm{CN}\;\left(f_{nk};\;{\hat{b}}_{nk}\left(i\right),\;v_{nk}^{b}\left(i\right)\right),$$

where $v_{nk}^{b}\left(i\right)$ and ${\hat{b}}_{nk}$ are given in [25] (Sec. II-D–Sec. II-E).

(53) $$v_{nk}^{b}\left(i\right)={\left(\sum_{m=1}^{M}{\left|{\hat{w}}_{mn}\left(i\right)\right|}^{2}v_{mk}^{\zeta }\left(i\right)\right)}^{-1},$$

(54) $${\hat{b}}_{nk}=\left(1-v_{nk}^{b}\left(i\right)\sum_{m=1}^{M}v_{mn}^{w}\left(i\right)v_{mk}^{\zeta }\left(i\right)\right){\hat{f}}_{nk}\left(i\right)+v_{nk}^{b}\left(i\right)\sum_{m=1}^{M}{\hat{w}}_{mn}\left(i\right){\hat{\zeta }}_{mk}\left(i\right).$$

In (54), we define the auxiliary variables as follows:

(55) $$v_{mk}^{\zeta }\left(i\right)=\frac{v_{mk}^{p}\left(i\right)-v_{mk}^{z}\left(i\right)}{{\left(v_{mk}^{p}\left(i\right)\right)}^{2}},\;{\hat{\zeta }}_{mk}\left(i\right)=\frac{{\hat{z}}_{mk}\left(i\right)-{\hat{p}}_{mk}\left(i\right)}{v_{mk}^{p}\left(i\right)}.$$

where $v_{mk}^{p}\left(i\right)$ and ${\hat{p}}_{mk}\left(i\right)$ are given as follows in [25] (Sec. II-F).

(56) $$v_{mk}^{p}\left(i\right)=\sum_{n=1}^{N^{\prime }}\left({\left|{\hat{w}}_{mn}\left(i\right)\right|}^{2}v_{nk}^{f}\left(i\right)+v_{mn}^{w}\left(i\right){\left|{\hat{f}}_{nk}\left(i\right)\right|}^{2}+v_{mn}^{w}\left(i\right)\;v_{nk}^{f}\left(i\right)\right),$$

(57) $${\hat{p}}_{mk}\left(i\right)=\sum_{n=1}^{N^{\prime }}{\hat{w}}_{mn}\left(i\right){\hat{f}}_{nk}\left(i\right)-{\hat{\zeta }}_{mk}\left(i-1\right)\sum_{n=1}^{N^{\prime }}\left({\left|{\hat{w}}_{mn}\left(i\right)\right|}^{2}v_{nk}^{f}\left(i\right)+v_{mn}^{w}\left(i\right){\left|{\hat{f}}_{nk}\left(i\right)\right|}^{2}\right).$$

Similar to (42), we can approximate that $P_{f_{nk}}^{i}\left(f_{nk}\right)\approx \mathrm{CN}\left(f_{nk};{\hat{\mu }}_{nk}\left(i\right)+h_{1,nk},v_{nk}^{\mu }\left(i\right)\right)$, where the mean and the variance are further specified in (67) and (68). Therefore, we can obtain the mean and variance of $\Delta_{f_{nk}}^{i+1}\left(f_{nk}\right)$ as follows:

(58) $$v_{nk}^{f}\left(i+1\right)=\frac{v_{nk}^{\mu }\left(i\right)\;v_{nk}^{b}\left(i\right)}{v_{nl}^{\mu }\left(i\right)+v_{nk}^{b}\left(i\right)},$$

(59) $${\hat{f}}_{nk}\left(i+1\right)=\frac{v_{nk}^{\mu }\left(i\right)\;{\hat{b}}_{nk}\left(i\right)+v_{nk}^{b}\left(i\right)\;{\hat{\mu }}_{nk}\left(i\right)+v_{nk}^{b}\left(i\right)\;h_{1,nk}}{v_{nk}^{\mu }\left(i\right)+v_{nk}^{b}\left(i\right)}.$$

Next, we approximate the messages associated with the variable nodes $\left\{w_{mn}\right\}$ as follows:

(60) $$\prod_{k=1}^{K}\Delta_{zwg_{mk}\to w_{mn}}^{i}\;\left(w_{mn}\right)=\mathrm{CN}\;\left(w_{mn};\;{\hat{c}}_{mn}\left(i\right),\;v_{mn}^{c}\left(i\right)\right),$$

where $v_{mn}^{c}\left(i\right)$ and ${\hat{c}}_{mn}\left(i\right)$ are given as follows:

(61) $$v_{mn}^{c}\left(i\right)={\left(\sum_{k=1}^{K}{\left|{\hat{f}}_{nk}\left(i\right)\right|}^{2}v_{mk}^{\zeta }\left(i\right)\right)}^{-1},$$

(62) $${\hat{c}}_{mn}\left(i\right)=\left(1-v_{mn}^{c}\left(i\right)\sum_{k=1}^{K}v_{nk}^{f}\left(i\right)v_{mk}^{s}\left(i\right)\right){\hat{w}}_{mn}\left(i\right)+v_{mn}^{c}\left(i\right)\sum_{k=1}^{K}{\hat{f}}_{nk}\left(i\right){\hat{\zeta }}_{mk}\left(i\right).$$

Similarly, we can approximate that $P_{w_{mn}}^{i}\left(w_{mn}\right)\approx \mathrm{CN}\left(w_{mn};{\hat{\rho }}_{mn}\left(i\right)+h_{0,mn},v_{mn}^{\rho }\left(i\right)\right)$, where the mean and the variance are further specified in (77) and (78). Therefore, we can obtain the mean and variance of $\Delta_{w_{mn}}^{i+1}\left(w_{mn}\right)$ as follows:

(63) $$v_{mn}^{w}\left(i+1\right)=\frac{v_{mn}^{\rho }\left(i\right)\;v_{mn}^{c}\left(i\right)}{v_{mn}^{\rho }\left(i\right)+v_{mn}^{c}\left(i\right)},$$

(64) $${\hat{w}}_{mn}\left(i+1\right)=\frac{v_{mn}^{\rho }\left(i\right)\;{\hat{c}}_{mn}\left(i\right)+v_{mn}^{c}\left(i\right)\;{\hat{\rho }}_{mn}\left(i\right)+v_{mn}^{c}\left(i\right)\;h_{0,mn}}{v_{mn}^{\rho }\left(i\right)+v_{mn}^{c}\left(i\right)}.$$

Subsequently, we approximate the messages associated with the variable nodes $\left\{g_{n^{\prime }k}\right\}$. Similar to (41), we obtain

(65) $$\prod_{n^{\prime },k}\Delta_{g_{n^{\prime }k}\to fg_{nk}}^{i}\left(g_{n^{\prime }k}\right)=\mathrm{CN}\left(g_{n^{\prime }k};{\hat{\mu }}_{nk}\left(i\right),v_{nk}^{\mu }\left(i\right)\right),$$

(66) $$\prod_{n,k}\Delta_{fg_{nk}\to g_{n^{\prime }k}}^{i}\left(g_{n^{\prime }k}\right)=\mathrm{CN}\left(g_{n^{\prime }k};{\hat{d}}_{nk}\left(i\right),v_{nk}^{d}\left(i\right)\right),$$

where

(67) $$v_{nk}^{\mu }\left(i\right)=\sum_{n^{\prime }=1}^{N^{\prime }}\sum_{k=1}^{K}{\left|a_{R,n^{\prime }n}\right|}^{2}v_{n^{\prime }k}^{g}\left(i\right),$$

(68) $${\hat{\mu }}_{nk}\left(i\right)=\sum_{n^{\prime }=1}^{N^{\prime }}\sum_{k=1}^{K}a_{R,n^{\prime }n}{\hat{g}}_{n^{\prime }k}\left(i\right)-v_{nk}^{\mu }\left(i\right){\hat{\alpha }}_{nk}\left(i-1\right),$$

(69) $$v_{n^{\prime }k}^{d}\left(i\right)={\left(\sum_{n=1}^{N^{\prime }}\sum_{k=1}^{K}{\left|a_{R,n^{\prime }n}\right|}^{2}v_{nk}^{\alpha }\left(i\right)\right)}^{-1},$$

(70) $${\hat{d}}_{n^{\prime }k}\left(i\right)={\hat{g}}_{n^{\prime }k}+v_{nk}^{d}\left(i\right)\sum_{n=1}^{N^{\prime }}\sum_{k=1}^{K}a_{R,n^{\prime }n}{\hat{\alpha }}_{nk}\left(i\right).$$

In (69) and (70), we define

(71) $$v_{nk}^{\alpha }\left(i\right)={\left(v_{nk}^{\mu }\left(i\right)+v_{nk}^{b}\left(i\right)\right)}^{-1},$$

(72) $${\hat{\alpha }}_{nk}\left(i\right)=v_{nk}^{\alpha }\left(i\right)\left({\hat{b}}_{nk}\left(i\right)-h_{1,nk}-{\hat{\mu }}_{nk}\left(i\right)\right).$$

Therefore, plugging (66) into (37), we obtain $\Delta_{g_{n^{\prime }k}}^{i+1}\left(g_{n^{\prime }k}\right)\approx p\left(g_{n^{\prime }k}\right)\mathrm{CN}\left(g_{n^{\prime }k};{\hat{d}}_{nk}\left(i\right),v_{nk}^{d}\left(i\right)\right)$ and

(73) $${\hat{g}}_{n^{\prime }k}\left(i+1\right)=\int g_{n^{\prime }k}\Delta_{g_{n^{\prime }k}}^{i+1}\left(g_{n^{\prime }k}\right)\;dg_{n^{\prime }k},$$

(74) $$v_{n^{\prime }k}^{g}\left(i+1\right)=\int g_{n^{\prime }k}^{2}\;\Delta_{g_{n^{\prime }k}}^{i+1}\left(g_{n^{\prime }k}\right)\;dg_{n^{\prime }k}-{\left|{\hat{g}}_{n^{\prime }k}\left(i+1\right)\right|}^{2}.$$

Finally, we approximate the messages associated with the variable nodes $\left\{s_{m^{\prime }n^{\prime }}\right\}$ as follows:

(75) $$\prod_{m^{\prime },n^{\prime }}\Delta_{s_{m^{\prime }n^{\prime }}\to ws_{mn}}^{i}\left(s_{m^{\prime }n^{\prime }}\right)=\mathrm{CN}\left(s_{m^{\prime }n^{\prime }};{\hat{\rho }}_{mn}\left(i\right),v_{mn}^{\rho }\left(i\right)\right),$$

(76) $$\prod_{m,n}\Delta_{ws_{mn}\to s_{m^{\prime }n^{\prime }}}^{i}\left(s_{m^{\prime }n^{\prime }}\right)=\mathrm{CN}\left(s_{m^{\prime }n^{\prime }};{\hat{e}}_{mn}\left(i\right),v_{mn}^{e}\left(i\right)\right).$$

where

(77) $$v_{mn}^{\rho }\left(i\right)=\sum_{m^{\prime }=1}^{M^{\prime }}\sum_{n^{\prime }=1}^{N^{\prime }}{\left|a_{B,m^{\prime }m}\right|}^{2}v_{m^{\prime }n^{\prime }}^{s}\left(i\right){\left|a_{R,n^{\prime }n}\right|}^{2},$$

(78) $${\hat{\rho }}_{mn}\left(i\right)=\sum_{m^{\prime }=1}^{M^{\prime }}\sum_{n^{\prime }=1}^{N^{\prime }}a_{B,mm^{\prime }}{\hat{s}}_{m^{\prime }n^{\prime }}\left(i\right)a_{R,n^{\prime }n}-v_{mn}^{l}\left(i\right){\hat{\beta }}_{mn}\left(i-1\right),$$

(79) $$v_{m^{\prime }n^{\prime }}^{e}\left(i\right)={\left(\sum_{m=1}^{M}\sum_{n=1}^{N^{\prime }}{\left|a_{B,mm^{\prime }}\right|}^{2}v_{mn}^{\beta }\left(i\right){\left|a_{R,n^{\prime }n}\right|}^{2}\right)}^{-1},$$

(80) $${\hat{e}}_{m^{\prime }n^{\prime }}\left(i\right)={\hat{s}}_{m^{\prime }n^{\prime }}+v_{m^{\prime }n^{\prime }}^{e}\left(i\right)\sum_{m=1}^{M}\sum_{n=1}^{N^{\prime }}a_{B,mm^{\prime }}{\hat{\beta }}_{mn}\left(i\right)a_{R,n^{\prime }n}.$$

In (79) and (80), we define

(81) $$v_{mn}^{\beta }\left(i\right)={\left(v_{mn}^{\rho }\left(i\right)+v_{mn}^{c}\left(i\right)\right)}^{-1},$$

(82) $${\hat{\beta }}_{mn}\left(i\right)=v_{mn}^{\beta }\left(i\right)\left({\hat{c}}_{mn}\left(i\right)-h_{0,mn}-{\hat{\rho }}_{mn}\left(i\right)\right).$$

Similarly, by plugging (76) into (40), we obtain $\Delta_{s_{m^{\prime }n^{\prime }}}^{i+1}\left(s_{m^{\prime }n^{\prime }}\right)\approx p\left(s_{m^{\prime }n^{\prime }}\right)\mathrm{CN}\left(s_{m^{\prime }n^{\prime }};{\hat{e}}_{m^{\prime }n^{\prime }}\left(i\right),v_{m^{\prime }n^{\prime }}^{e}\left(i\right)\right)$ and

(83) $${\hat{s}}_{m^{\prime }n^{\prime }}\left(i+1\right)=\int s_{m^{\prime }n^{\prime }}\Delta_{s_{m^{\prime }n^{\prime }}}^{i+1}\left(s_{m^{\prime }n^{\prime }}\right)\;ds_{m^{\prime }n^{\prime }},$$

(84) $$v_{m^{\prime }n^{\prime }}^{s}\left(i+1\right)=\int s_{m^{\prime }n^{\prime }}^{2}\;\Delta_{s_{m^{\prime }n^{\prime }}}^{i+1}\left(s_{m^{\prime }n^{\prime }}\right)\;ds_{m^{\prime }n^{\prime }}-{\left|{\hat{s}}_{m^{\prime }n^{\prime }}\left(i+1\right)\right|}^{2}.$$

We present the resulting algorithm in Algorithm 1. In this work, we adopt an adaptive damping approach to improve the convergence and robustness of mBiGAMP [25]. Specifically, at each iteration, we smooth the updates of the variance and mean of *Z*, *W*, *F*, *S*, and *G* by combining the current and previous updates. We stop Algorithm 1 once it reaches $I_{\max}=300$ iterations, or when the difference between two consecutive iterates is less than a small positive threshold ϵ.
**Algorithm 1** The mBiGAMP algorithm.**Input:** Y, $A_{B}$, $A_{R}$$H_{0}$, $H_{1}$, X, $\tau_{N}$, $\lambda_{S}$, $\lambda_{G}$.**Initialize:**${\hat{s}}_{m^{\prime }n^{\prime }}\left(1\right)$ randomly drawn from $p\left(s_{m^{\prime }n^{\prime }}\right)$${\hat{g}}_{n^{\prime }k}\left(1\right)$ randomly drawn from $p\left(g_{n^{\prime },k}\right)$${\hat{w}}_{mn}\left(1\right)=h_{0,mn}+\sum_{m^{\prime }n^{\prime }}a_{B,mm^{\prime }}{\hat{s}}_{m^{\prime }n^{\prime }}\left(1\right)a_{R,n^{\prime }n}^{*}$${\hat{f}}_{nk}\left(1\right)=h_{1,nk}+\sum_{n^{\prime }k}a_{R,nn^{\prime }}{\hat{g}}_{n^{\prime },k}\left(1\right)$${\hat{z}}_{mk}\left(1\right)=\sum_{n}{\hat{w}}_{mn}\left(1\right){\hat{f}}_{nk}\left(1\right)$   **for** $i=1,2,\ldots ,I_{\max}$ **do**     For ∀m,k update ${\hat{z}}_{mk}$ and $v_{mk}^{z}$ by (48), (46), (47), (56), (57) and (45).     For ∀n,k update ${\hat{f}}_{nk}$ and $v_{nk}^{f}$ by (53), (54), (67) (68), (58) and (59).     For ∀m,n update ${\hat{w}}_{mn}$ and $v_{mn}^{w}$ by (61), (62), (77), (78), (63) and (64).     For $\forall \;n^{\prime },k$ update ${\hat{g}}_{n^{\prime }k}$ and $v_{n^{\prime }k}^{g}$ by (71), (72), (69), (70), (73) and (74).     For $\forall \;m^{\prime },n^{\prime }$ update ${\hat{s}}_{m^{\prime }n^{\prime }}$ and $v_{m^{\prime }n^{\prime }}^{s}$ by (81), (82), (79), (80), (83) and (84).     **If** a certain stopping criterion is met, **stop**   **End for****Output:** ${\hat{s}}_{mn}$ and ${\hat{g}}_{nk}$

### 3.4. Complexity Analysis

In this subsection, we summarize the total number of multiplications and additions for each variable node in each algorithm iteration for the proposed mBiGAMP algorithm. Each iteration of the mBiGAMP algorithm consists of five steps. The specific computational complexity of each step is as follows: the computational complexity of ${\hat{z}}_{mk}$ and $v_{mk}^{z}$ is O(MKL); the computational complexity of ${\hat{f}}_{nk}$ and $v_{nk}^{f}$ is $O(MKN^{\prime })$; the computational complexity of ${\hat{w}}_{mn}$ and $v_{mn}^{w}$ is $O(MKN^{\prime })$; the computational complexity of ${\hat{g}}_{n^{\prime }k}$ and $v_{n^{\prime }k}^{g}$ is $O(K^{2}{\left(N^{\prime }\right)}^{2})$; and the computational complexity of ${\hat{s}}_{m^{\prime }n^{\prime }}$ and $v_{m^{\prime }n^{\prime }}^{s}$ is $O(MM^{\prime }{\left(N^{\prime }\right)}^{2})$. Therefore, the computational complexity of each iteration of the algorithm is $O(MKL+2MKN^{\prime }+{\left(K^{2}+MM\right)}^{\prime }{\left(N^{\prime }\right)}^{2})$. The computational complexity of the algorithm shows that when $N^{\prime }≳\sqrt{MKL/\left(K^{2}+MM^{\prime }\right)}$, the algorithm’s computational complexity increases with the square of the number of IRS elements. By contrast, the PARAFAC algorithm, which is also capable of segmented channel estimation, incurs a computational complexity of $O(2N^{3}+4N^{2}P\left(M+K\right)+4MNKP)$, where *P* denotes the number of feasible IRS phase configurations. It can be seen that the computational complexity of the PARAFAC algorithm increases with the cube of the number of IRS elements. Consequently, for large-scale IRS deployments, our algorithm entails a lower computational complexity than PARAFAC.

## 4. Simulation Results

In this section, we present the computational simulation results of the proposed method’s performance. We have particularly simulated the normalized mean square error (NMSE) using the metrics $∥{\hat{H}}_{RB}-H_{RB}{∥}^{2}/∥H_{RB}{∥}^{2}\;\mathrm{and}\;∥{\hat{H}}_{UR}-H_{UR}{∥}^{2}/∥H_{UR}{∥}^{2}$. We conducted the simulations using MATLAB 2022a. Each NMSE curve equals the sample mean of 500 statistically independent Monte Carlo channel realizations, and the iterations terminated at the tolerance $\epsilon =10^{-4}$. For the large-scale fading model, we use the Close-In Free Space Reference Distance with Frequency-Dependent Path Loss Exponent (CIF) model proposed in [26] to match the path loss and shadow fading of the auxiliary transportation tunnel in coal mines. The specific expression is

(85) $$L\left(dB\right)=32.4+20\;\mathrm{lg}\;\left(\frac{f_{c}}{\mathrm{GHz}}\right)+\left(24.237-1.754\;\mathrm{lg}\;\left(\frac{f_{c}}{\mathrm{GHz}}\right)\right)\mathrm{lg}\;\left(\frac{d}{m}\right)+X_{\sigma_{c}}$$

where $f_{c}=5.8$ GHz is the operating frequency band; *d* is the transmission distance. We set the distance from user to IRS and from IRS to BS to 20 m and 30 m, respectively; $X_{\sigma }$ is shadow fading, which represents large-scale channel fluctuations and is a Gaussian random variable with zero mean and standard deviation of $\sigma_{c}=11.1$. Furthermore, to demonstrate the superiority of the proposed algorithm, we compared it with the current state-of-the-art methods, including the PARAFAC decomposition algorithm, the DS-OMP algorithm, and the traditional LS algorithm.

**Figure 3:**
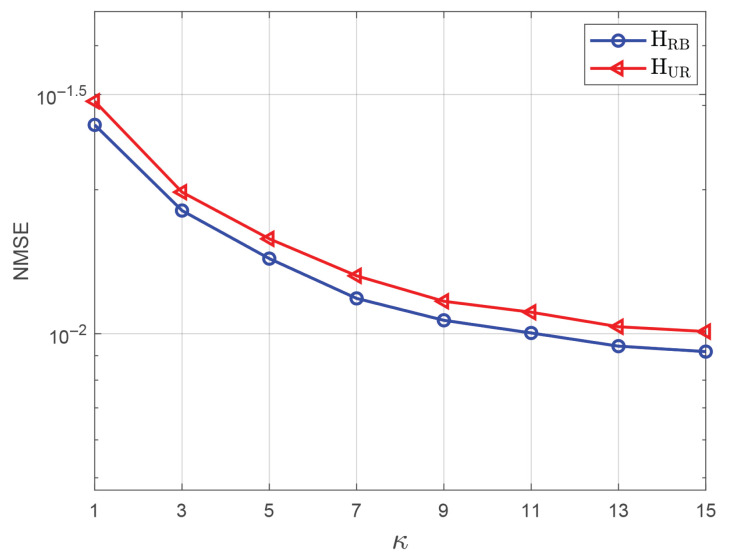
The NMSE performance versus Rician factor κ with SNR = 0 dB.

First, we investigated the impact of the Rician factor κ, used to control the fast-varying channel component, on the channel estimation performance, as shown in Figure 3. The parameter settings are $M^{\prime }=M=40$, $N_{1}=N_{1}^{\prime }=N_{2}=N_{2}^{\prime }=6$, K=36, $\lambda_{S}=\lambda_{G}=0.3$, L=40, and SNR = 0 dB. The parameter *m* in the Nakagami-*m* channel is set to 0.85. In the subsequent simulations, these parameters will remain unchanged unless otherwise stated. From Figure 3, it can be observed that as κ increases, the proportion of known information in the channel grows, which reduces the NMSE of the proposed mBiGAMP algorithm. Moreover, the NMSE decline rate diminishes with increasing κ. For convenience in the subsequent simulations, we uniformly set the value of κ to 9.

**Figure 4:**
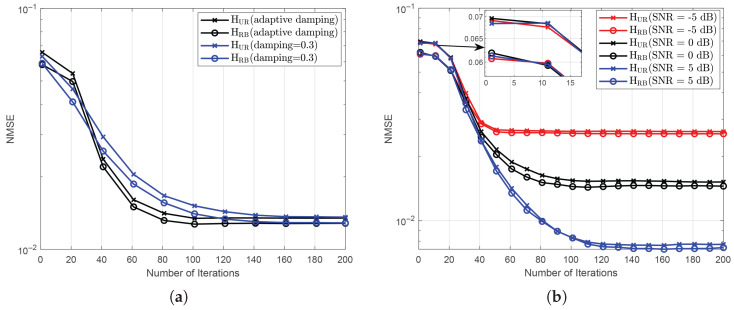
(**a**) Convergence performance comparison between adaptive damping and fixed damping factor; (**b**) The number of iterations of the mBiGAMP algorithm under different SNRs.

Next, to verify the superiority of adaptive damping, we compared it with the fixed damping factor β=0.3, as carefully selected in [27], as shown in Figure 4a. It can be seen that both methods converge as the number of iterations increases, but the adaptive damping approach achieves convergence in approximately 80 iterations, whereas the method with fixed β=0.3 requires about 120 iterations. To further validate the algorithm’s convergence under different SNR conditions, we examine the NMSE performance across varying iteration counts, as shown in Figure 4b. It is evident that our algorithm converges to different NMSE levels as the number of iterations increases.

**Figure 5:**
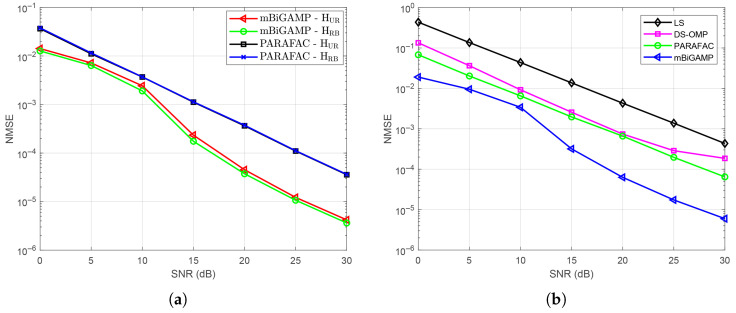
(**a**) NMSE performance of different separated channel estimation strategies versus SNR; (**b**) NMSE performance of different cascaded channel estimation strategies versus SNR.

Subsequently, we investigated the NMSE performance of the proposed mBiGAMP algorithm compared with the PARAFAC decomposition algorithm under separated channel estimation, as shown in Figure 5a. It can be observed that the mBiGAMP algorithm significantly outperforms the PARAFAC decomposition algorithm. At NMSE of $10^{-4}$, the proposed mBiGAMP algorithm achieves approximately a 7 dB gain over the PARAFAC decomposition algorithm, demonstrating its effectiveness for separated channel estimation. To further assess cascaded channel estimation, Figure 5b compares the NMSE achieved by the proposed mBiGAMP algorithm with those of the PARAFAC decomposition algorithm, the DS-OMP algorithm, and the LS algorithm. Similar performance improvements are observed as in the separated estimation case. Specifically, at NMSE of $10^{-3}$, the proposed mBiGAMP algorithm achieves gains of 5 dB, 6 dB, and 14 dB over the PARAFAC decomposition algorithm, DS-OMP algorithm, and LS algorithm, respectively.

**Figure 6:**
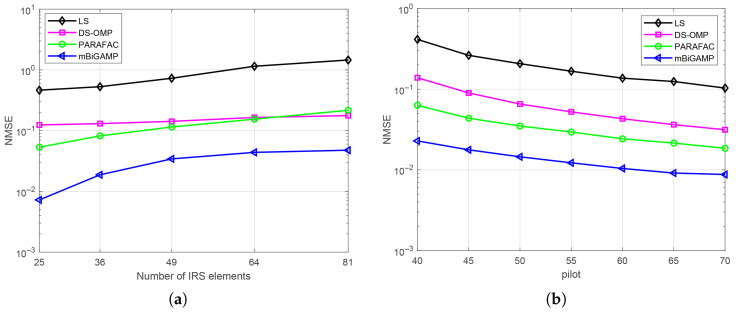
(**a**) Performance comparisons for different channel estimation algorithms with varying numbers of IRS elements; (**b**) Performance comparisons for different channel estimation algorithms with varying pilot lengths.

To further evaluate the performance of the proposed mBiGAMP algorithm under various system configurations, we conducted additional simulations for different numbers of IRS elements and varying pilot lengths as shown in Figure 6a. and Figure 6b. Figure 6a shows that as the IRS size increases from 5×5 to 9×9, the performance of all channel estimation algorithms degrades due to the increased number of estimated elements, leading to an accumulation of estimation errors. Despite this, the proposed mBiGAMP algorithm maintains superior performance compared to the other algorithms. Similarly, Figure 6b shows that all algorithms benefit from longer pilot length as the additional pilot symbols help improve estimation accuracy. Likewise, the proposed mBiGAMP algorithm consistently achieves better performances than the others with varying pilot lengths, which demonstrates that the proposed mBiGAMP algorithm achieves more accurate channel state information (CSI) recovery with lower pilot overhead while maintaining robust estimation accuracy.

**Figure 7:**
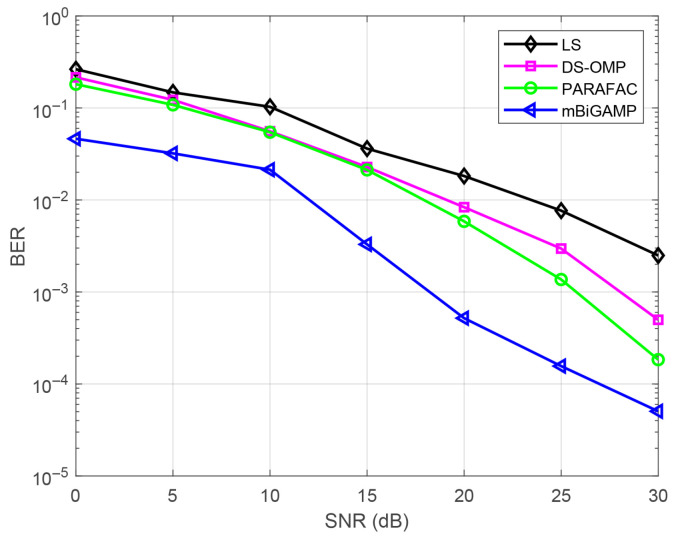
Comparison of BER performance of different channel estimation algorithms.

To better evaluate the practical impact of the proposed mBiGAMP algorithm, we further conduct signal detection on the channels estimated by different channel estimation algorithms to obtain the corresponding BER performance. We set the transmitted data length to be 10,000 and modulate them using QPSK. The BER result under different algorithms is shown in Figure 7. It can be observed that the proposed mBiGAMP algorithm consistently outperforms the other channel estimation algorithms. Specifically, when the NMSE is $10^{-2}$, mBiGAMP achieves SNR gains of approximately 6 dB, 7 dB, and 12 dB over PARAFAC, DS-OMP, and LS, respectively. These BER results provide a more comprehensive validation of the superiority of the proposed mBiGAMP algorithm over the other algorithms in the coal mine scenario.

**Figure 8:**
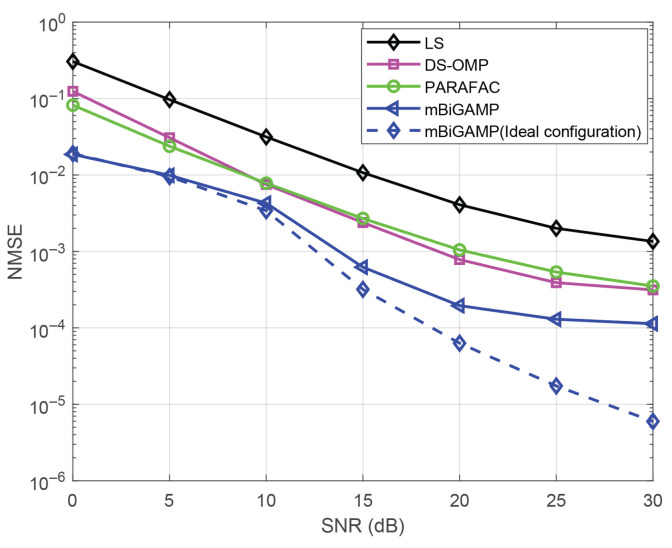
Comparison of performance of channel estimation algorithms under the same phase noise.

Finally, to verify the robustness of the proposed A algorithm, we analyze the case where phase noise exists in the IRS. As mentioned in [28], the IRS phase after adding noise is expressed as

(86) $$\Phi =\beta \;\mathrm{diag}\left(e^{j(\theta_{1}+\theta_{\sigma })},e^{j(\theta_{2}+\theta_{\sigma })},\ldots ,e^{j(\theta_{N}+\theta_{\sigma })}\right)$$

where β denotes the amplitude coefficient, θ is the ideal phase, and $\theta_{\sigma }$ is the introduced phase noise following the distribution of $N(0,\sigma_{\mathrm{IRS}}^{2})$. After introducing the same phase noise $\sigma_{\mathrm{IRS}}^{2}=1$, we compare the performance of the proposed mBiGAMP algorithm with other channel estimation algorithms, and the results are shown in Figure 8. It can be observed that the channel estimation performance degrades in the presence of phase noise, but the proposed mBiGAMP algorithm consistently outperforms other channel estimation algorithms under the same phase noise. Specifically, when the NMSE is $10^{-3}$, mBiGAMP achieves SNR gains of approximately 5 dB, 6 dB, and 16 dB over the DS-OMP, PARAFAC, and LS algorithms, respectively. These results show that our algorithm exhibits better robustness in the presence of phase noise compared to other algorithms.

## 5. Conclusions

In this paper, we proposed a mBiGAMP algorithm combined with IRS technology to enhance channel estimation accuracy in coal mine wireless communication systems. To accurately model the unique propagation environment characterized by abundant coal-carrying belt conveyors, we established a hybrid channel model incorporating both fast-varying and quasi-static components. Building upon this model, we introduced two additional factor nodes and variable nodes to capture distinct message-passing behaviors, thereby deriving the mBiGAMP algorithm. Simulation results show that the proposed mBiGAMP algorithm achieves substantial performance gains over other state-of-the-art algorithms in both separated and cascaded channel estimation in coal mines. Notably, the mBiGAMP algorithm exhibits robust convergence across the operational SNR range while requiring fewer pilot symbols to maintain estimation accuracy compared with other methods. Our work not only offers a feasible and efficient solution to improve channel estimation performance in coal mines, but it also provides a foundation for further research on IRS-assisted systems in confined and dynamic propagation scenarios.

## Figures and Tables

**Table 1:** Notations of means and variances for messages.

Message	Mean	Variance
$\Delta_{g_{n^{\prime }k}\to fg_{nk}}^{i}\left(g_{n^{\prime }k}\right)$	${\hat{g}}_{n^{\prime }k,nk}\left(i\right)$	$v_{n^{\prime }k,nk}^{g}\left(i\right)$
$\Delta_{s_{m^{\prime }n^{\prime }}\to ws_{mn}}^{i}\left(s_{m^{\prime }n^{\prime }}\right)$	${\hat{s}}_{m^{\prime }n^{\prime },mn}\left(i\right)$	$v_{m^{\prime }n^{\prime },mn}^{s}\left(i\right)$
$\Delta_{w_{mn}\to zwf_{mk}}^{i}\left(w_{mn}\right)$	${\hat{w}}_{mn,k}\left(i\right)$	$v_{mn,k}^{w}\left(i\right)$
$\Delta_{f_{nk}\to zwf_{mk}}^{i}\left(f_{nk}\right)$	${\hat{f}}_{nk,m}\left(i\right)$	$v_{nk,m}^{f}\left(i\right)$
$\Delta_{z_{mk}\to qz_{ml}}^{i}\left(z_{mk}\right)$	${\hat{z}}_{mk,l}\left(i\right)$	$v_{mk,l}^{z}\left(i\right)$
$\Delta_{g_{n^{\prime }k}}^{i}\left(g_{n^{\prime }k}\right)$	${\hat{g}}_{n^{\prime }k}\left(i\right)$	$v_{n^{\prime }k}^{g}\left(i\right)$
$\Delta_{s_{m^{\prime }n^{\prime }}}^{i}\left(s_{m^{\prime }n^{\prime }}\right)$	${\hat{s}}_{m^{\prime }n^{\prime }}\left(i\right)$	$v_{m^{\prime }n^{\prime }}^{s}\left(i\right)$
$\Delta_{w_{mn}}^{i}\left(w_{mn}\right)$	${\hat{w}}_{mn}\left(i\right)$	$v_{mn}^{w}\left(i\right)$
$\Delta_{f_{nk}}^{i}\left(f_{nk}\right)$	${\hat{f}}_{nk}\left(i\right)$	$v_{nk}^{f}\left(i\right)$
$\Delta_{z_{mk}}^{i}\left(z_{mk}\right)$	${\hat{z}}_{mk}\left(i\right)$	$v_{mk}^{z}\left(i\right)$

## Data Availability

Data are contained within the article.
